# Body mass index–related heterogeneity in diaphragm thickening fraction response to exercise training in stable chronic obstructive pulmonary disease

**DOI:** 10.3389/fphys.2026.1900140

**Published:** 2026-08-11

**Authors:** Yide Wang, Yidie Bao, Xinliao Deng, Hongxia Duan, Hui Li, Jiachi Zhang, Jian Li, Peijun Li, Xiaodan Liu

**Affiliations:** 1 Department of Rehabilitation, The Fourth Clinical Medical College of Xinjiang Medical University, Urumqi, China; 2 School of Rehabilitation Science, Shanghai University of Traditional Chinese Medicine, Shanghai, China; 3 Institute of Rehabilitation Medicine, Shanghai Academy of Traditional Chinese Medicine, Shanghai, China; 4 Engineering Research Center of Traditional Chinese Medicine Intelligent Rehabilitation, Ministry of Education, Shanghai, China

**Keywords:** body mass index, chronic obstructive pulmonary disease, diaphragm thickening fraction, exercise training, pulmonary rehabilitation

## Abstract

**Background:**

Diaphragmatic dysfunction contributes to exercise intolerance in COPD, but whether BMI shapes baseline function and response to exercise remains unclear. This secondary analysis characterized BMI-related diaphragmatic phenotypes and whether diaphragm thickening fraction (TFdi) links exercise training to functional improvement.

**Methods:**

Data were analyzed from 103 patients with stable COPD in a completed 2×2 factorial randomized trial. Because no exercise-by-acupuncture interaction was detected for 4-week TFdi, participants were regrouped as exercise-exposed or non-exercise. Associations between BMI and baseline TFdi were assessed using restricted cubic splines and threshold models. Regression models estimated exercise effects on 4-week TFdi and 6-minute walk distance (6MWD), with BMI-stratified interaction and exploratory mediation analyses.

**Results:**

BMI showed a nonlinear association with baseline TFdi, with an adjusted turning point at 23.7 kg/m². Below this threshold, BMI was positively associated with TFdi (β=0.05, 95% CI 0.03–0.08); above it, the association was negative (β=−0.05, 95% CI −0.07 to −0.03). Exercise was associated with higher 4-week TFdi (β=0.15, 95% CI 0.01–0.29) and longer 6MWD (β=59.61 m, 95% CI 10.65–108.57). The TFdi response was most pronounced at BMI ≥25.0 kg/m², with borderline interaction evidence. Exploratory mediation suggested TFdi partially mediated exercise-related 6MWD gains, particularly in this BMI group.

**Conclusions:**

This study integrates BMI phenotyping, diaphragm ultrasound, and mediation analysis to show that diaphragmatic function and exercise-induced diaphragmatic adaptation in COPD are BMI dependent. BMI may help identify patients most likely to derive diaphragmatic benefit from rehabilitation.

## Introduction

Chronic obstructive pulmonary disease (COPD) is a leading cause of morbidity and mortality worldwide (de Oca et al., 2025). Beyond airflow limitation, COPD is characterized by systemic manifestations, including dysfunction of both peripheral and respiratory muscles. Among these, diaphragmatic dysfunction is particularly important because chronic hyperinflation shortens and flattens the diaphragm, placing it at a mechanical disadvantage (Topcuoğlu et al., 2022). This impairment contributes to ventilatory limitation, exercise intolerance, and dyspnea. Ultrasound-derived diaphragm thickening fraction (TFdi) has emerged as a reliable, noninvasive marker of diaphragmatic contractile function in COPD, and lower TFdi has been associated with more severe airflow limitation, reduced six-minute walk distance (6MWD), and greater dyspnea (Rittayamai et al., 2020; An et al., 2022). These findings underscore the clinical relevance of preserving inspiratory muscle function in this population.

Body mass index (BMI) may be an important modifier of respiratory muscle structure and function in COPD. Low BMI is a recognized marker of muscle wasting and poor prognosis, whereas overweight and mild obesity have been associated with better survival, a phenomenon often described as the “obesity paradox.” However, this apparent benefit does not extend to extreme obesity, suggesting that the relationship between BMI and COPD severity is likely non-linear. Obesity is common in COPD and has been linked to greater symptom burden, reduced exercise capacity, and more frequent exacerbations in some studies, whereas others have reported no clear exercise disadvantage compared with lean patients (Shin et al., 2022; Wang et al., 2024). Such inconsistency may partly reflect obesity-related alterations in respiratory mechanics, including higher resting inspiratory capacity relative to total lung capacity and increased abdominal pressure, which may partially offset lung hyperinflation. Together, these observations support the existence of BMI-related COPD phenotypes, ranging from an underweight phenotype characterized by muscle wasting and impaired diaphragmatic force generation to an overweight phenotype with relatively preserved diaphragmatic function. Consistent with this concept, recent ultrasound studies have reported a positive correlation between TFdi and BMI in stable COPD (Schulz et al., 2022).

Exercise training is a cornerstone of pulmonary rehabilitation in COPD and improves exercise tolerance, symptoms, and quality of life (Blervaque et al., 2021). These benefits are thought to arise from multiple physiological adaptations, including reduced ventilatory demand and dynamic hyperinflation, improved oxygen delivery, and enhanced peripheral and respiratory muscle performance (Cao et al., 2022). Recent evidence further suggests that pulmonary rehabilitation can improve diaphragmatic function, as reflected by increases in TFdi and diaphragm excursion, and that greater diaphragmatic adaptation may be associated with larger gains in 6MWD (Ran et al., 2025). However, responses to exercise training vary substantially across individuals, and it remains unclear whether diaphragmatic adaptation differs according to BMI. Given that obesity may preserve muscle reserve whereas low BMI is associated with muscle loss, BMI may influence the magnitude of diaphragmatic improvement after exercise training. Whether training-induced changes in TFdi are also related to improvements in functional exercise capacity has not been well established.

Therefore, in this secondary analysis of a factorial randomized trial in stable COPD, we investigated BMI-related heterogeneity in diaphragmatic response to exercise training. Specifically, we examined whether exercise-induced changes in TFdi differed across BMI categories and whether patients with obesity exhibited greater diaphragmatic improvement. We also explored whether changes in TFdi were associated with improvements in 6MWD. By linking BMI-related COPD phenotypes with diaphragmatic plasticity and rehabilitation outcomes, this study may help clarify individual variation in response to exercise training in COPD.

## Methods

### Study design and data source

This study was a secondary analysis of a completed 2×2 factorial randomized controlled trial conducted in patients with stable chronic obstructive pulmonary disease (COPD). The parent trial included four intervention combinations: exercise plus acupuncture, acupuncture alone, exercise alone, and neither intervention (Duan et al., 2025). The parent trial was registered with the Chinese Clinical Trial Registry (ChiCTR; registration number: ChiCTR2300076255; trial record: https://www.chictr.org.cn/hvshowproject.html?id=247978&v=1.1) on 28 September 2023. This study is a secondary analysis of the registered parent trial, and no new participant recruitment, randomization, or intervention was performed for the present analysis.

For the present analysis, our primary aim was to investigate whether exercise improved diaphragmatic function, as assessed by diaphragm thickening fraction (TFdi), whether this effect was modified by body mass index (BMI), and whether improvement in TFdi might mediate the beneficial effect of exercise on exercise capacity. Because the parent trial used a factorial design, we first tested the exercise-by-acupuncture interaction on 4-week TFdi. As no significant interaction was detected, participants were subsequently collapsed into an exercise-exposed group (exercise alone and exercise plus acupuncture) and a non-exercise group (acupuncture alone and neither intervention) to estimate the average treatment effect of exercise. The current secondary analysis was therefore structured around four objectives: to characterize the baseline association between BMI and TFdi, to estimate the effect of exercise on 4-week TFdi, to examine BMI as an effect modifier of the exercise–TFdi association, and to explore TFdi as a potential mediator of exercise-related improvement in 6-minute walk distance (6MWD).

### Study population and eligibility criteria

A total of 103 patients with stable COPD from the parent randomized trial were included in this secondary analysis. Eligibility criteria were those of the parent trial, and no additional inclusion or exclusion criteria were introduced specifically for the present study. Thus, the analytic cohort corresponded to the original randomized population. For descriptive analyses, all 103 participants were considered. For regression-based complete-case analyses, participants were included if the relevant outcome and covariate data were available for the specific model being fitted. To evaluate baseline comparability after regrouping by exercise exposure, demographic and clinical characteristics were compared between the exercise and non-exercise groups. These baseline variables included age, sex, BMI, COPD duration, smoking history and smoking exposure, allergic history, hypertension, diabetes, coronary heart disease, asthma, and current COPD-related medications.

### Variable definitions

BMI was analyzed both as a continuous exposure and as a categorical variable. BMI categories were initially defined according to both the conventional international classification (underweight, <18.5 kg/m²; normal weight, 18.5–24.9 kg/m²; overweight, 25.0–29.9 kg/m²; and obesity, ≥30.0 kg/m²) and the Asia-Pacific classification (underweight, <18.5 kg/m²; normal weight, 18.5–22.9 kg/m²; overweight/at risk, 23.0–24.9 kg/m²; obesity class I, 25.0–29.9 kg/m²; and obesity class II, ≥30.0 kg/m²) (Lim et al., 2017; Okawa et al., 2025). Given the limited sample size, the original categories were collapsed for categorical analyses to reduce sparse-data bias and preserve statistical power. Accordingly, the international classification was grouped as non-overweight (<25 kg/m²) and overweight/obesity (≥25 kg/m²), whereas the Asia-Pacific classification was grouped as underweight/normal-weight (<23.0 kg/m²), overweight/at risk (23.0–24.9 kg/m²), and ≥25.0 kg/m² (corresponding to obesity class I/II in the Asia-Pacific classification). Because the study population was from the Asia-Pacific region, the Asia-Pacific classification was used as the primary stratification scheme for effect-modification and mediation analyses. Diaphragmatic ultrasonography provided end-expiratory diaphragmatic thickness, end-inspiratory diaphragmatic thickness, and TFdi, with TFdi calculated as (end-inspiratory thickness − end-expiratory thickness)/end-expiratory thickness. Baseline TFdi was used for phenotypic analyses, and 4-week TFdi was the primary post-intervention diaphragmatic endpoint. For mediation analysis, exercise assignment was treated as the exposure, TFdi measured at the end of treatment as the mediator, and 6MWD measured at the end of follow-up (4 weeks after treatment) as the outcome in order to strengthen temporal ordering. Prespecified covariates for adjusted analyses included age, sex, smoking exposure (pack-years), COPD duration, COPD severity as measured by FEV1% predicted, hypertension, diabetes, coronary heart disease, asthma, and current key COPD medications. Baseline values of the corresponding outcome were additionally included in adjusted models for post-intervention outcomes.

### Statistical analysis

Baseline characteristics were summarized using mean (standard deviation), median (interquartile range), or number (percentage), as appropriate. Categorical baseline variables were compared using the chi-square test or Fisher’s exact test. To describe the baseline BMI–TFdi phenotype, TFdi distributions across BMI categories were compared using the Mann–Whitney U test for two-group comparisons and the Kruskal–Wallis test for three-group comparisons. We then fitted subgroup-specific linear regression models with BMI as the independent variable and baseline TFdi as the dependent variable. Three models were used: Model 1, unadjusted; Model 2, adjusted for age and sex; and Model 3, further adjusted for smoking exposure, COPD duration, FEV1% predicted, hypertension, diabetes, coronary heart disease, asthma, and current key COPD medications. To assess nonlinearity, BMI was further modeled using restricted cubic splines, followed by piecewise linear threshold models to estimate the inflection point and the slopes below and above that point. Linear and threshold models were compared using likelihood ratio tests. A supplementary sensitivity analysis stratified participants at the inflection point identified in the fully adjusted model to assess directional consistency of the BMI–TFdi association across clinical subgroups rather than to formally test heterogeneity.

For treatment-effect analyses, linear regression models were used to estimate the association of exercise with 4-week TFdi and with 6MWD. Before collapsing the original four groups, the exercise-by-acupuncture interaction on 4-week TFdi was examined in sequential models. Thereafter, the primary treatment-effect models included an unadjusted model and a fully adjusted model; the latter adjusted for the baseline value of the corresponding outcome and the prespecified covariates listed above. Effect modification by BMI was formally evaluated by adding an exercise-by-BMI group interaction term to the model. Because interaction testing generally has lower statistical power, a two-sided P value <0.10 was prespecified as the threshold for potential effect modification, whereas a two-sided P<0.05 was used for main effects (Prochaska et al., 2026; Zhang et al., 2026). The primary analysis was based on complete cases. As a sensitivity analysis, missing values were handled using multiple imputation with a Markov chain Monte Carlo approach to generate five imputed datasets, and pooled estimates, standard errors, and confidence intervals were obtained across imputations. Finally, an exploratory mediation analysis was performed to decompose the total effect of exercise on 6MWD into direct and indirect effects through TFdi. Total, direct, and indirect effects were estimated using nonparametric bootstrap resampling with 1,000 repetitions and percentile-based 95% confidence intervals; the mediation proportion was calculated as the indirect effect divided by the total effect. Mediation analyses were conducted in an unadjusted model and a fully adjusted model that included baseline TFdi, baseline 6MWD, and the same covariates as above. All analyses were performed in R version 4.1.0 and RStudio version 1.4.1717.

## Results

### Baseline characteristics of the study population

A total of 103 patients with stable COPD were included in this secondary analysis, comprising 58 patients in the control group and 45 patients in the exercise group. In the observed dataset, the overall mean age was 67.07 ± 7.48 years, 71.84% were male, the mean body mass index was 23.24 ± 3.43 kg/m², and the median COPD duration was 4.00 years (IQR, 1.00–10.00 years). Because some baseline categorical variables contained missing values, percentages were calculated using non-missing observations for each variable. No statistically significant between-group differences were observed in age, sex, body mass index, COPD duration, smoking-related variables, history of allergy, comorbidities, or baseline medication use in the observed dataset (all P > 0.05) (**Supplementary Table 1**). After multiple imputation, the distributions of baseline variables remained largely consistent with those of the observed data, and all between-group comparisons remained non-significant (all P > 0.05) (**Table 1**), suggesting that multiple imputation did not materially distort the baseline profile of the study population and indicating that baseline characteristics were generally comparable between groups.

**Table 1:** Baseline characteristics of participants included in this secondary analysis after multiple imputation.

Characteristic	Overall (n = 103)	Control group (n = 58)	Exercise group (n = 45)	P value
Age, years	67.07 ± 7.52	68.10 ± 6.89	65.73 ± 8.15	0.113
Sex, n (%)				0.303
Male	74 (71.84)	44 (75.86)	30 (66.67)	
Female	29 (28.16)	14 (24.14)	15 (33.33)	
Body mass index, kg/m²	23.26 ± 3.44	23.14 ± 3.77	23.42 ± 3.00	0.679
COPD duration, years	4.00 (1.00–10.00)	5.50 (1.00–10.00)	3.00 (1.00–11.00)	0.667
Smoking history, n (%)				0.373
Yes	60 (58.25)	36 (62.07)	24 (53.33)	
No	43 (41.75)	22 (37.93)	21 (46.67)	
Smoking duration, years*	40.00 (25.00–41.50)	40.00 (23.75–42.00)	30.00 (26.25–40.00)	0.491
Cigarettes per day*	20.00 (19.00–40.00)	20.00 (20.00–33.75)	25.00 (17.00–40.00)	0.705
History of allergy, n (%)				0.127
Yes	33 (32.04)	15 (25.86)	18 (40.00)	
No	70 (67.96)	43 (74.14)	27 (60.00)	
Hypertension, n (%)				0.184
Yes	34 (33.01)	16 (27.59)	18 (40.00)	
No	69 (66.99)	42 (72.41)	27 (60.00)	
Diabetes mellitus, n (%)				0.146
Yes	9 (8.74)	3 (5.17)	6 (13.33)	
No	94 (91.26)	55 (94.83)	39 (86.67)	
Coronary heart disease, n (%)				0.264
Yes	8 (7.77)	3 (5.17)	5 (11.11)	
No	95 (92.23)	55 (94.83)	40 (88.89)	
Asthma, n (%)				0.963
Yes	7 (6.80)	4 (6.90)	3 (6.67)	
No	96 (93.20)	54 (93.10)	42 (93.33)	
Bronchodilator use at baseline, n (%)				0.148
Yes	76 (73.79)	46 (79.31)	30 (66.67)	
No	27 (26.21)	12 (20.69)	15 (33.33)	
Inhaled corticosteroid use at baseline, n (%)				0.442
Yes	11 (10.68)	5 (8.62)	6 (13.33)	
No	92 (89.32)	53 (91.38)	39 (86.67)	
Expectorant use at baseline, n (%)				0.713
Yes	8 (7.77)	5 (8.62)	3 (6.67)	
No	95 (92.23)	53 (91.38)	42 (93.33)	
Traditional Chinese medicine use at baseline, n (%)				0.793
Yes	42 (40.78)	23 (39.66)	19 (42.22)	
No	61 (59.22)	35 (60.34)	26 (57.78)	

Data are presented as mean ± standard deviation, median (interquartile range), or n (%). Continuous variables were compared using the independent-samples *t* test or Mann-Whitney U test, as appropriate. Categorical variables were compared using the chi-square test or Fisher’s exact test, as appropriate; Fisher’s exact test was used when expected cell counts were small.

*Calculated among participants with a smoking history. COPD, chronic obstructive pulmonary disease.

Bronchodilators included short-acting or long-acting bronchodilators, specifically short-acting β2-agonists, long-acting β2-agonists, short-acting muscarinic antagonists, and long-acting muscarinic antagonists. Corticosteroids refer to inhaled corticosteroids. Expectorants refer to commonly used mucoactive agents. Traditional Chinese medicine included decoctions, granules, and Chinese patent medicines.

### Association of BMI with baseline TFdi

Baseline TFdi showed different patterns across BMI categories. According to the international BMI classification, TFdi was lower in the overweight/obesity group than in the non-overweight group [0.26 (IQR, 0.12–0.43) vs. 0.38 (IQR, 0.28–0.56)], although the difference was not statistically significant (P = 0.227). Under the Asia-Pacific BMI classification, baseline TFdi differed significantly across categories (P = 0.030), with the highest median value in the overweight/at-risk group [0.44 (IQR, 0.39–0.59)] and the lowest in the obesity group [0.25 (IQR, 0.12–0.40)] (**Supplementary Table 2**). In stratified linear regression analyses, BMI was positively associated with baseline TFdi in the lower-BMI strata, whereas the association became negative or tended to be negative in the higher-BMI strata across the three models (**Supplementary Table 3**). These findings suggest a potentially non-linear association between BMI and baseline TFdi.

### Non-linear association between BMI and baseline TFdi

**Figure 1:**
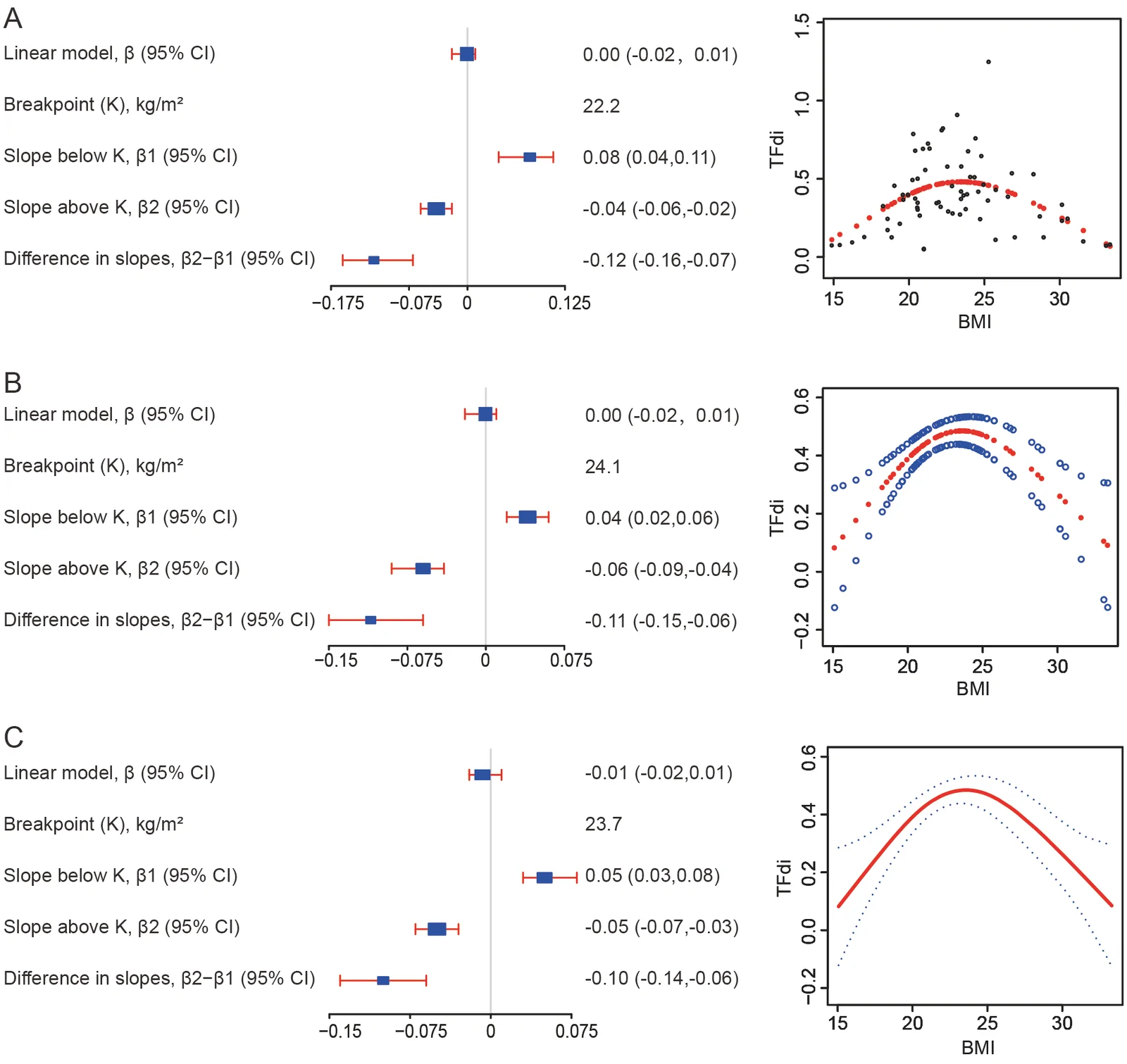
Restricted cubic spline plots of the association between BMI and baseline TFdi. Restricted cubic spline plots of the association between BMI and baseline TFdi under three regression models: **(A)** Model 1, unadjusted; **(B)** Model 2, adjusted for age and sex; and **(C)** Model 3, further adjusted for smoking exposure (pack-years), COPD duration, COPD severity (FEV1% predicted), hypertension, diabetes mellitus, coronary heart disease, coexisting asthma, and current key COPD medications. In each panel, the solid line represents the fitted association between BMI and TFdi, and the shaded area indicates the 95% confidence interval. The vertical dashed line indicates the estimated turning point from the corresponding threshold model: 22.2 kg/m² in **(A)**, 24.1 kg/m² in **(B)**, and 23.7 kg/m² in **(C)**. BMI, body mass index; TFdi, diaphragmatic thickening fraction; CI, confidence interval; COPD, chronic obstructive pulmonary disease.

The spline plots and threshold analyses jointly supported a non-linear association between BMI and baseline TFdi (**Figures 1A–C**). Although the overall linear association between BMI and TFdi was not statistically significant in any of the three models, the threshold model fit the data significantly better than the corresponding linear model in all models (all likelihood ratio test P < 0.001). The estimated turning points were 22.2 kg/m² in Model 1, 24.1 kg/m² in Model 2, and 23.7 kg/m² in Model 3. In the fully adjusted model, each 1 kg/m² increase in BMI was associated with a 0.05 increase in TFdi below 23.7 kg/m² (95% CI, 0.03 to 0.08; P < 0.0001) and a 0.05 decrease in TFdi above 23.7 kg/m² (95% CI, -0.07 to -0.03; P < 0.0001), with a significant difference between slopes (β2−β1 = -0.10, 95% CI, -0.14 to -0.06; P < 0.0001). Exploratory subgroup analyses stratified by the 23.7 kg/m² turning point showed broadly consistent directions of association across most estimable subgroups, providing supportive evidence for the robustness of the main findings (**Supplementary Table 4**).

### Interaction between exercise and acupuncture on 4-week TFdi

There was no evidence of a statistically significant interaction between exercise and acupuncture on 4-week TFdi in any of the three models (*P* for interaction = 0.809, 0.742, and 0.699, respectively) (**Supplementary Table 5**). In the fully adjusted model, none of the three intervention combinations differed significantly from the exercise-plus-acupuncture reference group. These findings supported the subsequent estimation of the average treatment effect of exercise on 4-week TFdi after regrouping participants according to exercise status.

### Effect of exercise on diaphragmatic function and modification by BMI

**Figure 2:**
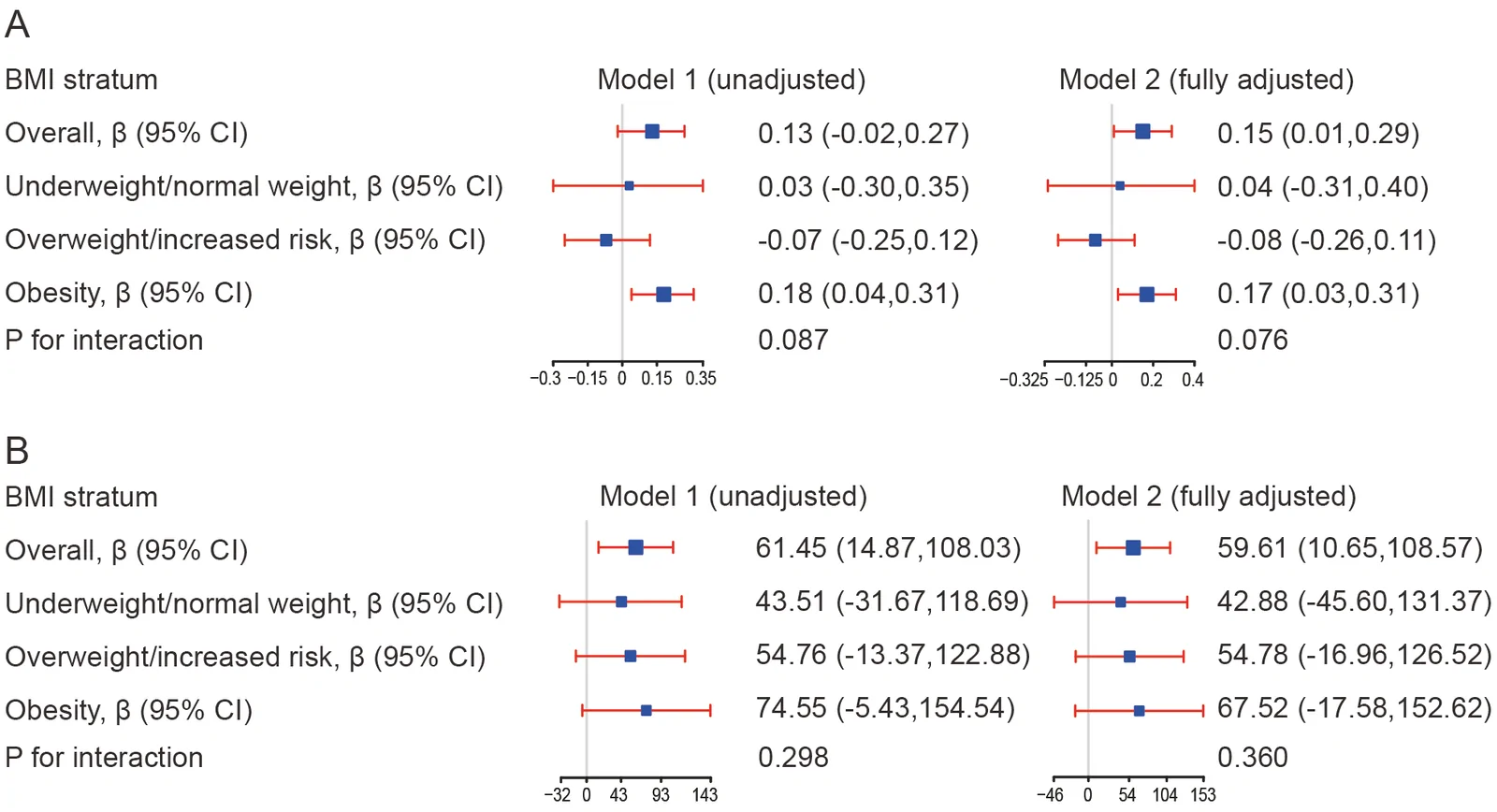
Forest plots of the association of exercise with 4-week diaphragm thickening fraction and 6-minute walk distance overall and across Asia-Pacific body mass index strata. Forest plots of the association of exercise with 4-week diaphragm thickening fraction [TFdi; **(A)**] and 6-minute walk distance [6MWD; **(B)**] in the overall sample and across Asia-Pacific body mass index (BMI) strata: underweight/normal weight (<23.0 kg/m²), overweight/increased risk (23.0–24.9 kg/m²), and BMI ≥25.0 kg/m² under the Asia-Pacific classification. Squares denote β coefficients and horizontal bars denote 95% confidence intervals. Model 1 was unadjusted. Model 2 was adjusted for the baseline value of the corresponding outcome, age, sex, smoking exposure (pack-years), COPD duration, COPD severity (FEV1% predicted), hypertension, diabetes mellitus, coronary heart disease, coexisting asthma, and current key COPD medications. *P* for interaction refers to the exercise × BMI-stratum interaction term. Exercise was coded as allocation to an exercise-containing group versus a non-exercise group; positive β values indicate higher TFdi or longer 6MWD in the exercise group. BMI, body mass index; CI, confidence interval; COPD, chronic obstructive pulmonary disease; TFdi, diaphragm thickening fraction; 6MWD, 6-minute walk distance.

In the complete-case analysis, exercise was positively associated with 4-week TFdi in the overall sample, and this association became statistically significant after full adjustment (β = 0.15, 95% CI 0.01 to 0.29; P = 0.041) (**Figure 2**). When stratified by BMI, the association was observed primarily in the ≥25.0 kg/m² group, in which exercise was associated with higher TFdi in both models, whereas no significant association was found in the <23.0 kg/m² or 23.0–24.9 kg/m² groups. Consistent with this pattern, the exercise × BMI-category interaction met the prespecified threshold for effect modification in both complete-case models (P for interaction = 0.087 and 0.076) and remained borderline in the fully adjusted multiple-imputation analysis (P = 0.097) (**Supplementary Table 6**). For EEDT, no clear association with exercise was observed in either the complete-case or multiple-imputation analyses. For EIDT, no significant overall association was observed in the complete-case analysis, whereas in the multiple-imputation analysis exercise was associated with higher EIDT in the fully adjusted model (β = 0.38 mm, 95% CI 0.02 to 0.74; P = 0.042), with a corresponding positive association in the ≥25.0 kg/m² group (β = 0.46 mm, 95% CI 0.14 to 0.79; P = 0.010), although no statistically significant interaction with BMI category was detected (**Supplementary Tables 7**, **8**). Overall, exercise showed the most consistent association with improvement in TFdi, with the largest estimated benefit observed in participants with obesity.

### Effect of exercise on 6MWD and modification by BMI

In the complete-case analysis, exercise was significantly associated with a longer 6MWD in the overall sample in both the unadjusted model (β = 61.45 m, 95% CI 14.87 to 108.03; P = 0.011) and the fully adjusted model (β = 59.61 m, 95% CI 10.65 to 108.57; P = 0.019) (**Figure 2**). Although point estimates were numerically larger in higher BMI categories, none of the BMI-specific associations reached statistical significance, and no significant interaction between exercise and BMI category was detected (P for interaction = 0.298 and 0.360 for Models 1 and 2, respectively). Results after multiple imputation were generally consistent, with a significant overall association retained in the fully adjusted model (β = 57.63 m, 95% CI 8.11 to 107.15; P = 0.025) but no statistically significant effect modification by BMI (**Supplementary Table 9**). Overall, these findings indicate that exercise was associated with improved walking capacity in stable COPD, without formal statistical evidence that the effect differed by BMI category.

### Effects of exercise on secondary outcomes beyond diaphragmatic function and 6MWD

Across secondary outcomes beyond diaphragmatic function and 6MWD, exercise showed the most consistent overall associations with lower SGRQ Activity scores, higher FEV1, lower rates of mild AECOPD, and lower NLRP3 levels, whereas most other outcomes were not significantly associated with exercise (**Supplementary Tables 10**-**16**). Exercise was also associated with higher MIP in the unadjusted model, but this association was attenuated after full adjustment. Formal interaction tests for BMI were generally non-significant, although exploratory signals of effect modification were observed for HAMA, MMEF25–75, FVC, and MEP in the fully adjusted models. Overall, these findings suggest that, beyond improving diaphragmatic function and walking capacity, exercise may additionally benefit activity limitation, selected spirometric indices, mild exacerbation risk, and NLRP3-related inflammation, whereas evidence for consistent BMI-dependent heterogeneity across these secondary outcomes remains limited.

### Exploratory mediation of the association between exercise and 6MWD through TFdi

**Figure 3:**
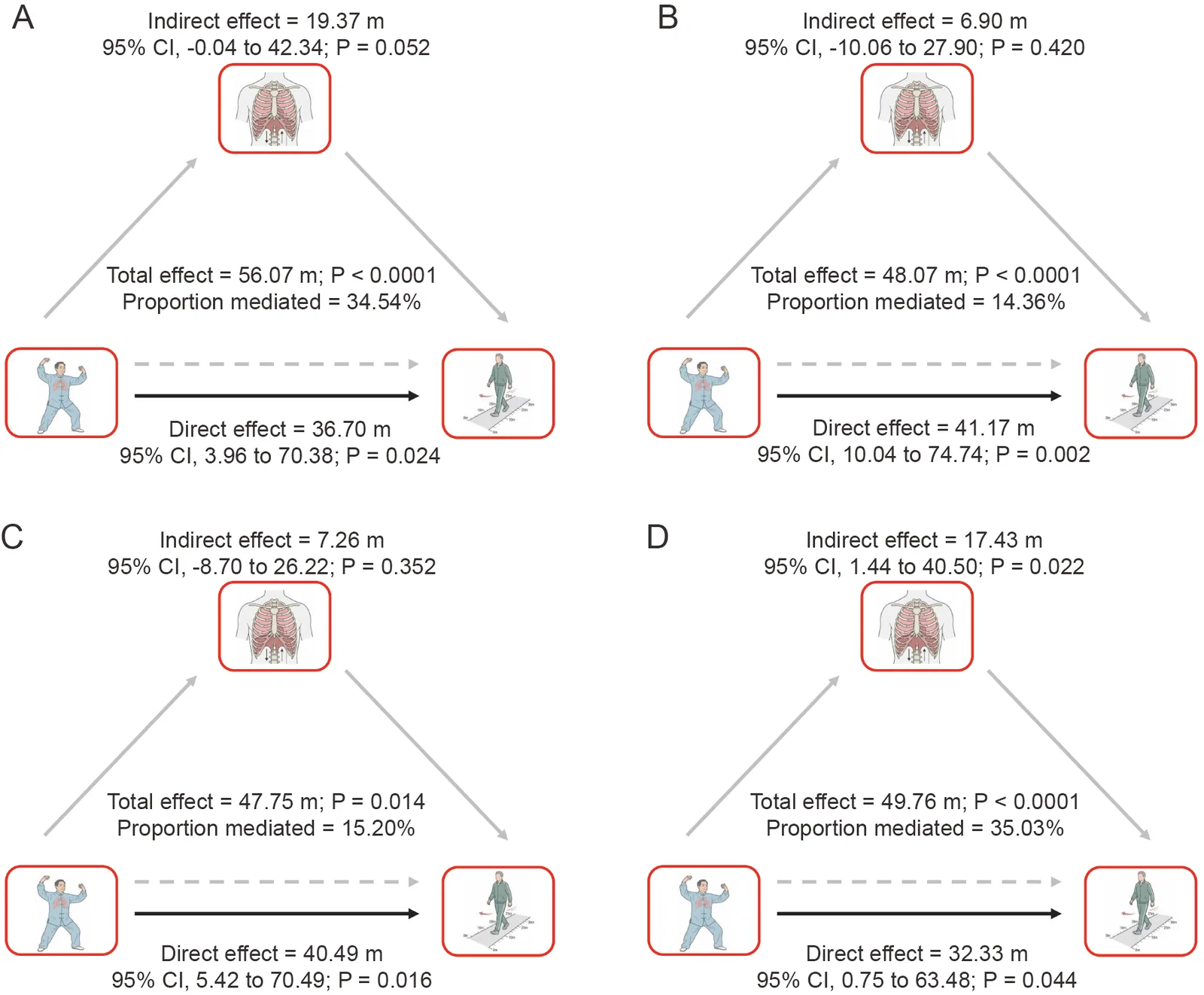
Fully adjusted mediation analyses of the association between exercise and 6-minute walk distance through diaphragm thickening fraction overall and by Asia-Pacific body mass index category. **(A)** shows the overall analysis; **(B)**, participants with BMI <23.0 kg/m² (underweight/normal-weight); **(C)**, participants with BMI 23.0–24.9 kg/m² (overweight/at risk); and **(D)**, participants with BMI ≥25.0 kg/m² (corresponding to obesity classes I/II in the Asia-Pacific classification). In each panel, exercise assignment was treated as the exposure, TFdi measured at the end of treatment as the mediator, and 6MWD measured at the end of follow-up as the outcome. Numbers shown are the estimated total, direct, and indirect effects (in meters) with 95% confidence intervals and P values from the fully adjusted mediation model; the proportion mediated is also presented. The fully adjusted model included baseline TFdi, baseline 6MWD, age, sex, smoking exposure (pack-years), COPD duration, COPD severity (FEV1% predicted), hypertension, diabetes mellitus, coronary heart disease, coexisting asthma, and current key COPD medications. Solid black arrows indicate direct effects, gray diagonal arrows indicate the indirect pathway through TFdi, and the dashed gray horizontal arrow denotes the overall association between exercise and 6MWD. TFdi was calculated as (end-inspiratory diaphragmatic thickness − end-expiratory diaphragmatic thickness)/end-expiratory diaphragmatic thickness. BMI, body mass index; TFdi, diaphragm thickening fraction; 6MWD, 6-minute walk distance; CI, confidence interval; COPD, chronic obstructive pulmonary disease.

Exploratory mediation analyses suggested that improvement in TFdi may partially mediate the association between exercise and improved 6MWD, with stronger signals observed in the ≥25.0 kg/m² group (**Figure 3**; **Supplementary Tables 17**, **18**). In the unadjusted model, the indirect effect via TFdi was statistically significant in the overall sample (19.02 m, 95% CI 1.16 to 37.45; P = 0.034), accounting for 29.37% of the total effect, whereas in the fully adjusted model the overall indirect effect was of borderline significance (19.37 m, 95% CI -0.04 to 42.34; P = 0.052). In contrast, a significant indirect effect remained evident in the ≥25.0 kg/m² group in both the unadjusted model (25.64 m, 95% CI 6.97 to 49.91; P = 0.002) and the fully adjusted model (17.43 m, 95% CI 1.44 to 40.50; P = 0.022), with estimated mediation proportions of 42.81% and 35.03%, respectively. No statistically significant indirect effects were observed in the <23.0 kg/m² or 23.0–24.9 kg/m² groups. Overall, these findings provide exploratory evidence consistent with partial mediation of the exercise-related improvement in walking capacity through TFdi, particularly among participants with obesity.

## Discussion

In this secondary analysis of a factorial randomized trial in stable COPD, we found that body mass index was related to diaphragmatic function in a non-linear rather than purely linear manner, and that exercise was associated with improved 4-week diaphragm thickening fraction (TFdi) after accounting for baseline values and relevant clinical covariates. The most notable pattern was that the exercise–TFdi association appeared to be stronger in participants with obesity, whereas comparable associations were not observed in the lower BMI strata. By contrast, although exercise improved 6-minute walk distance (6MWD) in the overall sample, we did not find formal statistical evidence that BMI modified the exercise effect on 6MWD itself. Exploratory mediation analyses further suggested that improvement in TFdi may explain part of the exercise-related gain in 6MWD, particularly in the obesity subgroup, although the overall adjusted indirect effect was only borderline significant. Taken together, these findings support a phenotype-oriented interpretation in which BMI is relevant not only to baseline diaphragmatic status but also, potentially, to the magnitude of diaphragmatic adaptation to exercise in stable COPD.

The baseline non-linear association between BMI and TFdi is biologically plausible. At lower BMI levels, increasing BMI may reflect better preservation of muscle mass and respiratory muscle reserve, whereas beyond a certain level of adiposity the relationship may reverse because excess body mass imposes mechanical disadvantages on diaphragmatic function (MaChado et al., 2023; Cesanelli et al., 2024; Tenda et al., 2024). In COPD, low BMI is closely linked to muscle wasting, sarcopenia, lower exercise capacity, and worse prognosis, whereas the interpretation of higher BMI is more complex (Keogh and Mark Williams, 2021; MaChado et al., 2021; Gómez-Martínez et al., 2023; Attaway et al., 2024). Epidemiologic and clinical studies have long suggested an “obesity paradox” in COPD, but this apparent advantage is neither universal nor mechanistically straightforward (Yohannes, 2022). Recent studies indicate that obesity may coexist with relatively preserved exercise capacity or spirometric indices in some COPD populations, while still altering respiratory mechanics, chest wall loading, and diaphragm geometry (Boriek et al., 2017; Lo Mauro et al., 2023). In this context, our findings suggest that moderate BMI may be relatively favorable for diaphragmatic contractile performance, whereas both low BMI and higher BMI may be associated with diaphragmatic disadvantage through different pathways. The estimated turning point around 23.7 kg/m² also strengthens the clinical interpretability of our results, because it lies close to the boundary between normal-weight and overweight/at-risk categories in the Asia-Pacific BMI classification used in our main effect-modification analyses.

The treatment findings likewise deserve careful interpretation. Before estimating the average treatment effect of exercise, we formally tested the exercise-by-acupuncture interaction on 4-week TFdi and found no evidence of a significant interaction, which supports the statistical rationale for collapsing the original four trial arms into exercise-exposed and non-exercise groups in this secondary analysis. Within this framework, exercise showed the most consistent association with TFdi rather than with static diaphragmatic thickness measures, suggesting that the principal signal may lie in dynamic diaphragmatic contractile behavior rather than resting thickness alone. This interpretation is consistent with recent literature showing that diaphragmatic ultrasound parameters, particularly thickening and excursion, are informative functional markers in COPD and may respond to rehabilitation (Shiraishi et al., 2020; Shiraishi et al., 2021; Ichiba et al., 2023; Yetkin et al., 2025). Pulmonary rehabilitation is well established as a core intervention for COPD and is associated with improvements in exercise capacity, dyspnea, and health status (Silva et al., 2022; Rochester et al., 2023; Vatrella et al., 2026). However, previous work indicates that respiratory muscle-directed strategies do not uniformly translate into additional 6MWD gains, even when inspiratory muscle strength improves (Sillen et al., 2022; Huang et al., 2024; Xie et al., 2025). Evidence directly addressing rehabilitation in patients with COPD and obesity remains limited. In a randomized single-blind trial restricted to 49 hospitalized patients with obesity and acute exacerbation of COPD, Torres-Sánchez et al. reported that adding a multimodal program comprising breathing training and upper- and lower-limb exercise to standard care improved lower-limb strength, exercise capacity, and selected quality-of-life and psychological outcomes (Torres-Sánchez et al., 2016). In a prospective multicenter cohort of 261 patients with stable COPD, Sava et al. found that pulmonary rehabilitation improved exercise capacity and health status across normal-weight, overweight, and obese groups, but that BMI category did not influence the magnitude of improvement (Sava et al., 2010). Neither study assessed diaphragm ultrasound outcomes. Our findings therefore extend this literature by suggesting a potentially diaphragm-specific pattern: the clearest estimated TFdi response occurred in the BMI ≥25.0 kg/m² subgroup, whereas BMI did not modify the exercise effect on 6MWD. Obesity-related changes in chest wall loading and diaphragm geometry could plausibly alter the capacity for diaphragmatic adaptation (Boriek et al., 2017; Lo Mauro et al., 2023), but this explanation remains speculative because body composition, abdominal adiposity, and dynamic hyperinflation were not directly measured. Moreover, the exercise-by-BMI interaction for TFdi met only the prespecified borderline threshold; therefore, the observed subgroup pattern should be regarded as hypothesis-generating rather than definitive evidence of treatment-effect heterogeneity.

The relationship between diaphragmatic improvement and exercise tolerance is particularly interesting. In our study, exercise improved 6MWD overall, but BMI did not significantly modify that overall functional effect. This suggests that the dominant pathway of exercise benefit may differ across BMI phenotypes. In participants with obesity, the exploratory mediation analysis suggested that improvement in TFdi may account for a meaningful proportion of the gain in 6MWD, whereas such a signal was not observed in the lower BMI strata. A cautious interpretation is that diaphragmatic recovery may be one relevant mechanism of exercise benefit in patients with obesity-related diaphragmatic disadvantage, but not necessarily the only or dominant mechanism in the broader COPD population. This interpretation is also consistent with prior IMT and pulmonary rehabilitation studies showing that respiratory muscle improvements may coexist with variable effects on functional exercise outcomes, depending on baseline physiological limitation, intervention duration, and concomitant peripheral adaptations (Pancera et al., 2025). Importantly, our secondary outcomes broadly support the same phenotype-oriented reading: exercise had the most consistent overall associations with TFdi, 6MWD, activity limitation, selected spirometric measures, mild AECOPD, and NLRP3, whereas evidence for BMI-dependent heterogeneity across most non-diaphragmatic secondary outcomes was limited. Accordingly, TFdi should be regarded as a plausible but still exploratory mechanism linking exercise to improved walking capacity, rather than a definitively established mediator.

This study has several strengths. It was conducted within a randomized trial framework, which improves internal validity for estimating the exercise effect. The parent trial used a factorial design, and the absence of a detectable exercise-by-acupuncture interaction strengthens the methodological legitimacy of focusing on the average treatment effect of exercise in the present secondary analysis. We also modeled BMI both continuously and categorically, which allowed us to detect a threshold-like baseline association rather than forcing a linear assumption. In addition, we performed multiple imputation and found that the principal treatment findings were directionally robust. Several limitations should also be acknowledged. First, this was a secondary analysis rather than a trial specifically designed to test BMI-related heterogeneity or mediation, so these aspects should be interpreted as *post hoc* and exploratory. Second, the sample size was modest, especially after BMI stratification, which limits statistical power for subgroup and interaction analyses and increases uncertainty around subgroup-specific estimates. Third, the intervention lasted only 4 weeks, which may be sufficient to detect short-term physiological change but not to define longer-term diaphragmatic adaptation. Fourth, BMI is a crude anthropometric index and does not distinguish fat mass from lean mass; direct body composition measures may provide more mechanistic resolution in future work. Finally, although we strengthened temporal ordering in the mediation analysis by using end-of-treatment TFdi and later 6MWD, the parent trial was not originally designed as a dedicated mechanistic study, and causal mediation inference therefore remains limited.

## Conclusion

From a clinical perspective, these findings argue against a one-size-fits-all view of respiratory adaptation in COPD rehabilitation. They suggest that diaphragmatic dysfunction is not uniformly distributed across body habitus and that patients with obesity may represent a subgroup in whom the diaphragm is both more impaired at baseline and more responsive to exercise-based rehabilitation. This does not imply that obesity is globally advantageous in COPD, nor does it imply that lower-BMI patients do not benefit from exercise; rather, it suggests that the dominant pathway of benefit may differ across phenotypes. In patients with obesity, diaphragmatic contractile adaptation may be a clinically relevant component of rehabilitation response, whereas in lower-BMI patients gains in exercise capacity may depend more on peripheral conditioning, symptom adaptation, or other physiological pathways. Future studies should validate these findings in larger and preferably multicenter cohorts; test continuous BMI-by-exercise interactions in adequately powered designs; incorporate direct body composition, hyperinflation, and respiratory muscle strength measurements; and determine whether diaphragm-focused strategies confer additional benefit in patients with obesity-related diaphragmatic impairment. Overall, our results support the view that the diaphragmatic response to exercise in stable COPD is heterogeneous and that BMI may serve as one clinically useful marker for identifying patients most likely to derive diaphragmatic benefit from rehabilitation.

## Data Availability

The raw data supporting the conclusions of this article will be made available by the authors, without undue reservation.
