# Supplementary material for: Body mass index–related heterogeneity in diaphragm thickening fraction response to exercise training in stable chronic obstructive pulmonary disease
Source: Front Physiol. 2026 Aug 11;17:1900140. doi: 10.3389/fphys.2026.1900140 (PMC13503601; doi:10.3389/fphys.2026.1900140)
Supplement: Supplementary file 1 [file Table1.docx]

**Supplementary Table 1. Baseline characteristics of participants included in this secondary analysis based on the observed data**

| **Characteristic** | **Overall** | **Control group (n = 58)** | **Exercise group (n = 45)** | **P value** |
| --- | --- | --- | --- | --- |
| Age, years | 67.07 ± 7.48 | 68.10 ± 6.89 | 65.73 ± 8.15 | 0.113 |
| Sex, n (%) |  |  |  | 0.303 |
| Male | 74 (71.84) | 44 (75.86) | 30 (66.67) |  |
| Female | 29 (28.16) | 14 (24.14) | 15 (33.33) |  |
| Body mass index, kg/m² | 23.24 ± 3.43 | 23.14 ± 3.77 | 23.42 ± 3.00 | 0.679 |
| COPD duration, years | 4.00 (1.00–10.00) | 5.50 (1.00–10.00) | 3.00 (1.00–11.00) | 0.764 |
| Smoking history, n (%) |  |  |  | 0.693 |
| Yes | 47 (52.22) | 26 (54.17) | 21 (50.00) |  |
| No | 43 (47.78) | 22 (45.83) | 21 (50.00) |  |
| Smoking duration, years* | 40.00 (25.00–41.50) | 40.00 (23.75–42.00) | 30.00 (26.25–40.00) | 0.841 |
| Cigarettes per day* | 20.00 (19.00–40.00) | 20.00 (20.00–33.75) | 25.00 (17.00–40.00) | 0.768 |
| History of allergy, n (%) |  |  |  | 0.122 |
| Yes | 32 (32.00) | 15 (25.86) | 17 (40.48) |  |
| No | 68 (68.00) | 43 (74.14) | 25 (59.52) |  |
| Hypertension, n (%) |  |  |  | 0.266 |
| Yes | 32 (32.00) | 16 (27.59) | 16 (38.10) |  |
| No | 68 (68.00) | 42 (72.41) | 26 (61.90) |  |
| Diabetes mellitus, n (%) |  |  |  | 0.221 |
| Yes | 8 (8.00) | 3 (5.17) | 5 (11.90) |  |
| No | 92 (92.00) | 55 (94.83) | 37 (88.10) |  |
| Coronary heart disease, n (%) |  |  |  | 0.207 |
| Yes | 6 (6.00) | 2 (3.45) | 4 (9.52) |  |
| No | 94 (94.00) | 56 (96.55) | 38 (90.48) |  |
| Asthma, n (%) |  |  |  | 0.306 |
| Yes | 5 (5.00) | 4 (6.90) | 1 (2.38) |  |
| No | 95 (95.00) | 54 (93.10) | 41 (97.62) |  |
| Bronchodilator use at baseline, n (%) |  |  |  | 0.181 |
| Yes | 66 (73.33) | 38 (79.17) | 28 (66.67) |  |
| No | 24 (26.67) | 10 (20.83) | 14 (33.33) |  |
| Inhaled corticosteroid use at baseline, n (%) |  |  |  | 0.573 |
| Yes | 9 (10.00) | 4 (8.33) | 5 (11.90) |  |
| No | 81 (90.00) | 44 (91.67) | 37 (88.10) |  |
| Expectorant use at baseline, n (%) |  |  |  | 0.891 |
| Yes | 4 (4.44) | 2 (4.17) | 2 (4.76) |  |
| No | 86 (95.56) | 46 (95.83) | 40 (95.24) |  |
| Traditional Chinese medicine use at baseline, n (%) |  |  |  | 0.353 |
| Yes | 34 (37.78) | 16 (33.33) | 18 (42.86) |  |
| No | 56 (62.22) | 32 (66.67) | 24 (57.14) |  |

Data are presented as mean ± standard deviation, median (interquartile range), or n (%). For variables with missing data, percentages for categorical variables were calculated using the number of non-missing observations for that variable as the denominator rather than the total sample size. In the observed dataset, the numbers of evaluable participants for smoking history, bronchodilator use, inhaled corticosteroid use, expectorant use, and traditional Chinese medicine use were 90 overall, 48 in the control group, and 42 in the exercise group. For history of allergy, hypertension, diabetes mellitus, coronary heart disease, and asthma, the number of evaluable participants was 100 overall. P values were calculated based on non-missing observations for each variable. Continuous variables were compared using the independent-samples *t* test or Mann-Whitney U test, as appropriate. Categorical variables were compared using the chi-square test or Fisher’s exact test, as appropriate.
*Calculated among participants with a smoking history.
Abbreviations: COPD, chronic obstructive pulmonary disease.

Bronchodilators included short-acting or long-acting bronchodilators, specifically short-acting β2-agonists, long-acting β2-agonists, short-acting muscarinic antagonists, and long-acting muscarinic antagonists. Corticosteroids refer to inhaled corticosteroids. Expectorants refer to commonly used mucoactive agents. Traditional Chinese medicine included decoctions, granules, and Chinese patent medicines.

**Supplementary Table 2. Baseline TFdi according to international and Asia-Pacific BMI classifications**

| **BMI classification** | **Subgroup** | **n** | **Baseline TFdi, median (IQR)** | **P value** |
| --- | --- | --- | --- | --- |
| International BMI classification | Non-overweight (<25 kg/m²) | 76 | 0.38 (0.28–0.56) | 0.227 |
|  | Overweight/obesity (≥25 kg/m²) | 27 | 0.26 (0.12–0.43) |  |
| Asia-Pacific BMI classification | Underweight/normal-weight (<23 kg/m²) | 49 | 0.35 (0.24–0.50) | 0.030 |
|  | Overweight/at-risk (23.0–24.9 kg/m²) | 27 | 0.44 (0.39–0.59) |  |
|  | Obesity (≥25.0 kg/m²) | 27 | 0.25 (0.12–0.40) |  |

Data are presented as median (interquartile range, IQR). Under the international BMI classification, participants were categorized as non-overweight (<25 kg/m²) or overweight/obesity (≥25 kg/m²). Under the Asia-Pacific BMI classification, participants were categorized as underweight/normal-weight (<23 kg/m²), overweight/at-risk (23.0–24.9 kg/m²), or obesity (≥25.0 kg/m²). Comparisons between two groups were performed using the Mann-Whitney U test, and comparisons among three groups were performed using the Kruskal-Wallis test. BMI, body mass index; TFdi, diaphragmatic thickening fraction.

**Supplementary Table 3. Stratified linear regression of the association between BMI and baseline TFdi within BMI categories**

| **BMI classification** | **Subgroup** | **n** | **Model 1, β (95% CI)** | **P value** | **Model 2, β (95% CI)** | **P value** | **Model 3, β (95% CI)** | **P value** |
| --- | --- | --- | --- | --- | --- | --- | --- | --- |
| International BMI classification | Non-overweight | 76 | 0.04 (0.02 to 0.07) | 0.0004 | 0.04 (0.02 to 0.07) | 0.0005 | 0.04 (0.02 to 0.07) | 0.0010 |
|  | Overweight/obesity | 27 | -0.06 (-0.10 to -0.01) | 0.0225 | -0.06 (-0.11 to 0.01) | 0.0518 | -0.07 (-0.13 to -0.01) | 0.0450 |
| Asia-Pacific BMI classification | Underweight/normal-weight | 49 | 0.06 (0.03 to 0.10) | 0.0008 | 0.06 (0.03 to 0.10) | 0.0013 | 0.06 (0.03 to 0.10) | 0.0015 |
|  | Overweight/at-risk | 27 | -0.01 (-0.18 to 0.15) | 0.8660 | -0.04 (-0.22 to 0.14) | 0.6526 | -0.04 (-0.22 to 0.14) | 0.6770 |
|  | Obesity | 27 | -0.06 (-0.11 to -0.01) | 0.0293 | -0.06 (-0.12 to 0.01) | 0.0640 | -0.07 (-0.14 to 0.01) | 0.0542 |

Stratified linear regression analyses were performed within each BMI subgroup, with BMI as the independent variable and baseline TFdi as the dependent variable. The β coefficient represents the change in TFdi associated with a 1 kg/m² increase in BMI within each subgroup. Model 1 was unadjusted. Model 2 was adjusted for age and sex. Model 3 was further adjusted for smoking exposure (pack-years), COPD duration, COPD severity (FEV1% predicted), hypertension, diabetes mellitus, coronary heart disease, coexisting asthma, and current key COPD medications. BMI, body mass index; CI, confidence interval; TFdi, diaphragmatic thickening fraction; COPD, chronic obstructive pulmonary disease.

**Supplementary Table 4. Exploratory subgroup analyses of the association between BMI and baseline TFdi stratified by the turning point identified in Model 3**

| **Subgroup variable** |  | **BMI <23.7 kg/m², n** | **β (95% CI)** | **P value** | **BMI ≥23.7 kg/m², n** | **β (95% CI)** | **P value** |
| --- | --- | --- | --- | --- | --- | --- | --- |
| Sex | Male | 38 | 0.05 (0.02 to 0.08) | 0.0059 | 36 | -0.05 (-0.08 to -0.01) | 0.0167 |
|  | Female | 21 | 0.07 (0.00 to 0.13) | 0.0536 | 9 | NE | NE |
| Age | <65 years | 20 | 0.08 (0.03 to 0.12) | 0.0072 | 19 | -0.06 (-0.17 to 0.05) | 0.3210 |
|  | ≥65 years | 39 | 0.05 (0.01 to 0.09) | 0.0214 | 26 | -0.03 (-0.06 to -0.01) | 0.0111 |
| COPD duration | <5 years | 34 | 0.07 (0.03 to 0.11) | 0.0030 | 19 | NE | NE |
|  | ≥5 years | 25 | 0.03 (-0.01 to 0.08) | 0.1371 | 26 | -0.04 (-0.08 to -0.00) | 0.0526 |
| History of allergy | Yes | 20 | 0.05 (0.00 to 0.10) | 0.0548 | 12 | NE | NE |
|  | No | 38 | 0.06 (0.02 to 0.10) | 0.0046 | 31 | -0.05 (-0.08 to -0.02) | 0.0081 |
| Smoking status | Yes | 23 | 0.05 (0.02 to 0.09) | 0.0156 | 24 | -0.03 (-0.06 to -0.01) | 0.0375 |
|  | No | 28 | 0.03 (-0.03 to 0.10) | 0.3482 | 16 | -0.05 (-0.07 to -0.02) | 0.0033 |
| Hypertension | Yes | 15 | NE | NE | 17 | NE | NE |
|  | No | 43 | 0.05 (0.02 to 0.09) | 0.0029 | 26 | -0.05 (-0.11 to 0.01) | 0.1566 |
| Diabetes mellitus | Yes | 4 | NE | NE | 4 | NE | NE |
|  | No | 54 | 0.06 (0.03 to 0.09) | 0.0006 | 39 | -0.05 (-0.08 to -0.02) | 0.0040 |
| Coronary heart disease | Yes | 4 | NE | NE | 2 | NE | NE |
|  | No | 54 | 0.06 (0.03 to 0.09) | 0.0010 | 41 | -0.05 (-0.08 to -0.02) | 0.0034 |
| Asthma | Yes | 4 | NE | NE | 1 | NE | NE |
|  | No | 54 | 0.05 (0.02 to 0.08) | 0.0011 | 42 | -0.05 (-0.08 to -0.02) | 0.0042 |
| Bronchodilator use | Yes | 36 | 0.06 (0.02 to 0.10) | 0.0068 | 31 | -0.04 (-0.07 to -0.02) | 0.0015 |
|  | No | 15 | NE | NE | 9 | NE | NE |
| Inhaled corticosteroid use | Yes | 6 | NE | NE | 3 | NE | NE |
|  | No | 45 | 0.04 (0.00 to 0.08) | 0.0414 | 37 | -0.04 (-0.06 to -0.03) | 0.0001 |
| Expectorant use | Yes | 4 | NE | NE | 0 | NE | NE |
|  | No | 47 | 0.05 (0.02 to 0.08) | 0.0050 | 40 | -0.04 (-0.06 to -0.03) | <0.0001 |
| Traditional Chinese medicine use | Yes | 19 | 0.03 (-0.01 to 0.07) | 0.1287 | 15 | -0.04 (-0.07 to -0.02) | 0.0054 |
|  | No | 32 | 0.06 (0.01 to 0.10) | 0.0255 | 25 | -0.05 (-0.07 to -0.02) | 0.0088 |

Exploratory subgroup analyses were performed after stratifying participants at the turning point identified in Model 3 (BMI = 23.7 kg/m²). Within each BMI stratum, BMI was entered as a continuous independent variable and baseline TFdi as the dependent variable. Results are presented as adjusted β coefficients with 95% confidence intervals. When a subgroup variable was itself included in the fully adjusted model, that variable was not additionally adjusted for in the corresponding subgroup analysis, whereas the remaining covariates were retained. Because of the limited sample size and sparse data in several subgroups after stratification, interaction P values were not calculated, and statistical significance within individual subgroups was not used as the primary basis for interpretation. NE indicates that a reliable estimate could not be obtained because of sparse data and/or model instability. BMI, body mass index; TFdi, diaphragmatic thickening fraction; CI, confidence interval.

**Supplementary Table 5. Interaction between exercise and acupuncture on 4-week TFdi**

| **Group** | **Model 1, β (95% CI)** | **Model 2, β (95% CI)** | **Model 3, β (95% CI)** |
| --- | --- | --- | --- |
| Exercise (+), Acupuncture (+) | Ref. | Ref. | Ref. |
| Exercise (-), Acupuncture (+) | -0.01 (-0.18 to 0.16) | -0.02 (-0.19 to 0.16) | -0.03 (-0.21 to 0.14) |
| Exercise (+), Acupuncture (-) | 0.08 (-0.07 to 0.23) | 0.07 (-0.08 to 0.23) | 0.08 (-0.08 to 0.23) |
| Exercise (-), Acupuncture (-) | 0.10 (-0.05 to 0.24) | 0.09 (-0.06 to 0.25) | 0.09 (-0.07 to 0.24) |
| *P* for interaction | 0.809 | 0.742 | 0.699 |

The outcome was TFdi at week 4. The exercise (+), acupuncture (+) group was used as the reference group. Model 1 was unadjusted. Model 2 was adjusted for baseline TFdi, age, and sex. Model 3 was further adjusted for smoking exposure (pack-years), COPD duration, COPD severity (FEV1% predicted), hypertension, diabetes mellitus, coronary heart disease, coexisting asthma, and current key COPD medications. *P* for interaction corresponds to the product term for exercise × acupuncture in each regression model and was used to assess whether the association between exercise and 4-week TFdi differed according to acupuncture status. CI, confidence interval; Ref., reference; TFdi, diaphragmatic thickening fraction; COPD, chronic obstructive pulmonary disease.

**Supplementary Table 6. Sensitivity analysis after multiple imputation: effect of exercise on 4-week TFdi overall and by BMI category**

| **Model** | **Overall, β (95% CI)** | **P value** | **<23 kg/m², β (95% CI)** | **P value** | **23.0–24.9 kg/m², β (95% CI)** | **P value** | **≥25.0 kg/m², β (95% CI)** | **P value** | **P for interaction** |
| --- | --- | --- | --- | --- | --- | --- | --- | --- | --- |
| Model 1 | 0.15 (0.00 to 0.30) | 0.055 | 0.03 (-0.30 to 0.35) | 0.863 | -0.07 (-0.25 to 0.12) | 0.503 | 0.16 (0.03 to 0.30) | 0.024 | 0.117 |
| Model 2 | 0.16 (0.01 to 0.30) | 0.037 | 0.01 (-0.32 to 0.35) | 0.942 | -0.08 (-0.27 to 0.11) | 0.405 | 0.16 (0.03 to 0.30) | 0.026 | 0.097 |

Values are β coefficients with 95% confidence intervals and represent the estimated effect of exercise intervention relative to non-exercise intervention on TFdi at week 4; a positive β indicates a higher TFdi in the exercise group. Model 1 was unadjusted. Model 2 was fully adjusted for baseline TFdi, age, sex, smoking exposure (pack-years), COPD duration, COPD severity (FEV1% predicted), hypertension, diabetes mellitus, coronary heart disease, coexisting asthma, and current key COPD medications. BMI categories were defined according to the Asia-Pacific criteria (<23.0, 23.0–24.9, and ≥25.0 kg/m²). *P* for interaction corresponds to the exercise × BMI-category interaction term. Missing data were handled using multiple imputation. TFdi, diaphragmatic thickening fraction; CI, confidence interval.

**Supplementary Table 7. Complete-case analysis of the effect of exercise on EEDT and EIDT overall and by BMI category**

| **Outcome** | **Model** | **Overall, β (95% CI), mm** | **P value** | **<23 kg/m², β (95% CI), mm** | **P value** | **23.0–24.9 kg/m², β (95% CI), mm** | **P value** | **≥25.0 kg/m², β (95% CI), mm** | **P value** | **P for interaction** |
| --- | --- | --- | --- | --- | --- | --- | --- | --- | --- | --- |
| EEDT | Model 1 | -0.02 (-0.27 to 0.22) | 0.847 | 0.03 (-0.72 to 0.78) | 0.933 | -0.10 (-0.53 to 0.34) | 0.676 | -0.02 (-0.31 to 0.27) | 0.906 | 0.170 |
|  | Model 2 | 0.05 (-0.16 to 0.26) | 0.641 | 0.30 (-0.42 to 1.02) | 0.428 | -0.15 (-0.58 to 0.29) | 0.521 | 0.04 (-0.20 to 0.27) | 0.762 | 0.194 |
| EIDT | Model 1 | 0.21 (-0.12 to 0.55) | 0.213 | 0.37 (-0.48 to 1.21) | 0.415 | -0.32 (-1.03 to 0.38) | 0.381 | 0.38 (-0.02 to 0.78) | 0.074 | 0.875 |
|  | Model 2 | 0.26 (-0.06 to 0.58) | 0.115 | 0.68 (-0.15 to 1.51) | 0.138 | -0.42 (-1.10 to 0.26) | 0.250 | 0.38 (-0.01 to 0.75) | 0.055 | 0.653 |

Values are β coefficients with 95% confidence intervals and represent the estimated effect of exercise intervention relative to non-exercise intervention on diaphragmatic thickness at week 4; a positive β indicates greater thickness in the exercise group. Model 1 was unadjusted. Model 2 was fully adjusted for age, sex, smoking exposure (pack-years), COPD duration, COPD severity (FEV1% predicted), hypertension, diabetes mellitus, coronary heart disease, coexisting asthma, and current key COPD medications; baseline EEDT was additionally adjusted for analyses of EEDT, and baseline EIDT was additionally adjusted for analyses of EIDT. BMI categories were defined according to the Asia-Pacific criteria (<23.0, 23.0–24.9, and ≥25.0 kg/m²). *P* for interaction corresponds to the exercise × BMI-category interaction term. EEDT, end-expiratory diaphragmatic thickness; EIDT, end-inspiratory diaphragmatic thickness; CI, confidence interval.

**Supplementary Table 8. Sensitivity analysis after multiple imputation: effect of exercise on EEDT and EIDT overall and by BMI category**

| **Outcome** | **Model** | **Overall, β (95% CI), mm** | **P value** | **<23 kg/m², β (95% CI), mm** | **P value** | **23.0–24.9 kg/m², β (95% CI), mm** | **P value** | **≥25.0 kg/m², β (95% CI), mm** | **P value** | **P for interaction** |
| --- | --- | --- | --- | --- | --- | --- | --- | --- | --- | --- |
| EEDT | Model 1 | 0.00 (-0.24 to 0.24) | 0.984 | 0.12 (-0.63 to 0.86) | 0.765 | -0.02 (-0.40 to 0.36) | 0.924 | -0.02 (-0.31 to 0.28) | 0.920 | 0.228 |
|  | Model 2 | 0.14 (-0.07 to 0.36) | 0.187 | 0.41 (-0.12 to 0.94) | 0.175 | -0.04 (-0.47 to 0.38) | 0.852 | 0.17 (-0.04 to 0.38) | 0.128 | 0.312 |
| EIDT | Model 1 | 0.23 (-0.10 to 0.56) | 0.177 | 0.45 (-0.40 to 1.30) | 0.320 | -0.24 (-0.91 to 0.44) | 0.507 | 0.36 (-0.05 to 0.77) | 0.098 | 0.665 |
|  | Model 2 | 0.38 (0.02 to 0.74) | 0.042 | 0.94 (-0.07 to 1.82) | 0.073 | -0.13 (-0.88 to 0.62) | 0.746 | 0.46 (0.14 to 0.79) | 0.010 | 0.717 |

Values are β coefficients with 95% confidence intervals and represent the estimated effect of exercise intervention relative to non-exercise intervention on diaphragmatic thickness at week 4; a positive β indicates greater thickness in the exercise group. Model 1 was unadjusted. Model 2 was fully adjusted for age, sex, smoking exposure (pack-years), COPD duration, COPD severity (FEV1% predicted), hypertension, diabetes mellitus, coronary heart disease, coexisting asthma, and current key COPD medications; baseline EEDT was additionally adjusted for analyses of EEDT, and baseline EIDT was additionally adjusted for analyses of EIDT. BMI categories were defined according to the Asia-Pacific criteria (<23.0, 23.0–24.9, and ≥25.0 kg/m²). *P* for interaction corresponds to the exercise × BMI-category interaction term. Missing data were handled using multiple imputation. EEDT, end-expiratory diaphragmatic thickness; EIDT, end-inspiratory diaphragmatic thickness; CI, confidence interval.

**Supplementary Table 9. Sensitivity analysis after multiple imputation: effect of exercise on 6MWD overall and by BMI category**

| **Stratum** | **Model 1, β (95% CI), m; P value** | **Model 2, β (95% CI), m; P value** |
| --- | --- | --- |
| Overall | 46.23 (-0.42 to 92.03); 0.052 | 57.63 (8.11 to 107.15); 0.025 |
| <23.0 kg/m² | 14.39 (-80.94 to 109.72); 0.772 | 44.60 (-40.76 to 129.97); 0.319 |
| 23.0–24.9 kg/m² | 48.52 (-16.96 to 114.00); 0.164 | 50.35 (-20.11 to 120.81); 0.177 |
| ≥25.0 kg/m² | 69.00 (-10.50 to 148.50); 0.098 | 61.49 (-26.84 to 149.82); 0.180 |
| *P* for interaction | 0.302 | 0.283 |

Values are β coefficients with 95% confidence intervals and represent the estimated effect of exercise intervention relative to non-exercise intervention on 6MWD; a positive β indicates a longer walking distance in the exercise group. Model 1 was unadjusted. Model 2 was fully adjusted for baseline 6MWD, age, sex, smoking exposure (pack-years), COPD duration, COPD severity (FEV1% predicted), hypertension, diabetes mellitus, coronary heart disease, coexisting asthma, and current key COPD medications. BMI categories were defined according to the Asia-Pacific criteria (<23.0, 23.0–24.9, and ≥25.0 kg/m²). *P* for interaction corresponds to the exercise × BMI-category interaction term. Missing data were handled using multiple imputation. 6MWD, 6-minute walk distance; CI, confidence interval.

**Supplementary Table 10. Effect of exercise on SGRQ total score and domain scores overall and by BMI category**

| **Outcome / Stratum** | **Model 1, β (95% CI), points; P value** | **Model 2, β (95% CI), points; P value** |
| --- | --- | --- |
| **SGRQ total score** |  |  |
| Overall | -5.41 (-11.52 to 0.69); 0.086 | -5.43 (-11.83 to 0.98); 0.101 |
| <23.0 kg/m² | -3.96 (-13.61 to 5.69); 0.426 | -4.14 (-14.54 to 6.27); 0.441 |
| 23.0–24.9 kg/m² | -3.85 (-15.66 to 7.96); 0.530 | -3.37 (-17.04 to 10.29); 0.635 |
| ≥25.0 kg/m² | -9.71 (-19.60 to 0.19); 0.068 | -10.18 (-20.34 to 0.01); 0.065 |
| *P* for interaction | 0.626 | 0.617 |
| **Symptoms** |  |  |
| Overall | 1.32 (-5.67 to 8.32); 0.711 | 2.21 (-4.96 to 9.38); 0.548 |
| <23.0 kg/m² | -0.20 (-10.22 to 9.81); 0.969 | 0.85 (-9.62 to 11.32); 0.875 |
| 23.0–24.9 kg/m² | 3.74 (-12.41 to 19.89); 0.655 | 6.56 (-11.33 to 24.46); 0.482 |
| ≥25.0 kg/m² | 1.78 (-9.94 to 13.51); 0.768 | 1.67 (-10.65 to 13.98); 0.794 |
| *P* for interaction | 0.775 | 0.699 |
| **Activity** |  |  |
| Overall | -10.44 (-19.26 to -1.63); 0.023 | -11.61 (-20.88 to -2.33); 0.016 |
| <23.0 kg/m² | -9.57 (-23.63 to 4.48); 0.189 | -10.91 (-25.95 to 4.13); 0.163 |
| 23.0–24.9 kg/m² | -5.56 (-20.76 to 9.63); 0.481 | -7.50 (-25.01 to 10.02); 0.413 |
| ≥25.0 kg/m² | -16.96 (-32.73 to -1.18); 0.047 | -17.97 (-33.79 to -2.15); 0.038 |
| *P* for interaction | 0.433 | 0.505 |
| **Impacts** |  |  |
| Overall | -4.74 (-11.17 to 1.68); 0.151 | -4.39 (-11.12 to 2.33); 0.204 |
| <23.0 kg/m² | -1.98 (-12.15 to 8.19); 0.705 | -1.87 (-12.81 to 9.07); 0.739 |
| 23.0–24.9 kg/m² | -5.45 (-17.64 to 6.73); 0.390 | -4.43 (-18.63 to 9.77); 0.549 |
| ≥25.0 kg/m² | -9.23 (-19.79 to 1.32); 0.101 | -9.50 (-20.55 to 1.55); 0.108 |
| *P* for interaction | 0.685 | 0.596 |

Values are β coefficients with 95% confidence intervals and represent the estimated effect of exercise intervention relative to non-exercise intervention on SGRQ scores; negative β values indicate lower scores and better respiratory health status in the exercise group. Model 1 was unadjusted. Model 2 was fully adjusted for age, sex, smoking exposure (pack-years), COPD duration, COPD severity (FEV1% predicted), hypertension, diabetes mellitus, coronary heart disease, coexisting asthma, and current key COPD medications; baseline SGRQ total score was additionally adjusted for the total score analysis, and the corresponding baseline domain score was adjusted for each domain-specific analysis. BMI categories were defined according to the Asia-Pacific criteria (<23.0, 23.0–24.9, and ≥25.0 kg/m²). *P* for interaction corresponds to the exercise × BMI-category interaction term. SGRQ, St. George’s Respiratory Questionnaire; CI, confidence interval.

**Supplementary Table 11. Effect of exercise on symptom burden scores overall and by BMI category**

| **Outcome / Stratum** | **Model 1, β (95% CI), points; P value** | **Model 2, β (95% CI), points; P value** |
| --- | --- | --- |
| **mMRC** |  |  |
| Overall | -0.36 (-0.76 to 0.05); 0.088 | -0.39 (-0.81 to 0.04); 0.076 |
| <23.0 kg/m² | -0.13 (-0.83 to 0.58); 0.724 | -0.11 (-0.92 to 0.71); 0.799 |
| 23.0–24.9 kg/m² | -0.47 (-1.14 to 0.20); 0.186 | -0.46 (-1.17 to 0.24); 0.212 |
| ≥25.0 kg/m² | -0.42 (-1.07 to 0.24); 0.221 | -0.47 (-1.18 to 0.24); 0.204 |
| *P* for interaction | 0.457 | 0.774 |
| **CAT** |  |  |
| Overall | 0.41 (-0.46 to 1.28); 0.355 | 0.53 (-0.35 to 1.40); 0.243 |
| <23.0 kg/m² | 0.68 (-1.14 to 2.51); 0.472 | 0.64 (-1.00 to 2.28); 0.454 |
| 23.0–24.9 kg/m² | 0.63 (-0.92 to 2.18); 0.434 | 0.64 (-0.99 to 2.28); 0.450 |
| ≥25.0 kg/m² | 0.13 (-1.16 to 1.42); 0.842 | 0.08 (-1.28 to 1.43); 0.912 |
| *P* for interaction | 0.514 | 0.715 |

Values are β coefficients with 95% confidence intervals and represent the estimated effect of exercise intervention relative to non-exercise intervention on symptom scores; negative β values indicate lower symptom burden in the exercise group. Model 1 was unadjusted. Model 2 was fully adjusted for age, sex, smoking exposure (pack-years), COPD duration, COPD severity (FEV1% predicted), hypertension, diabetes mellitus, coronary heart disease, coexisting asthma, and current key COPD medications; baseline mMRC and baseline CAT were additionally adjusted for the corresponding analyses. BMI categories were defined according to the Asia-Pacific criteria (<23.0, 23.0–24.9, and ≥25.0 kg/m²). *P* for interaction corresponds to the exercise × BMI-category interaction term. mMRC, modified Medical Research Council dyspnea scale; CAT, COPD Assessment Test; CI, confidence interval.

**Supplementary Table 12. Effect of exercise on anxiety and depression scores overall and by BMI category**

| **Outcome / Stratum** | **Model 1, β (95% CI), points; P value** | **Model 2, β (95% CI), points; P value** |
| --- | --- | --- |
| **HAMA** |  |  |
| Overall | -0.28 (-0.95 to 0.38); 0.406 | -0.25 (-0.95 to 0.46); 0.495 |
| <23.0 kg/m² | 0.16 (-0.18 to 0.49); 0.371 | 0.19 (-0.18 to 0.56); 0.311 |
| 23.0–24.9 kg/m² | -0.18 (-0.83 to 0.47); 0.591 | -0.24 (-0.86 to 0.38); 0.463 |
| ≥25.0 kg/m² | -1.21 (-3.64 to 1.22); 0.340 | -1.42 (-4.10 to 1.25); 0.312 |
| *P* for interaction | 0.104 | 0.079 |
| **HAMD** |  |  |
| Overall | -0.02 (-0.88 to 0.84); 0.968 | 0.01 (-0.90 to 0.91); 0.988 |
| <23.0 kg/m² | 0.97 (-0.33 to 2.26); 0.150 | 1.14 (-0.26 to 2.54); 0.119 |
| 23.0–24.9 kg/m² | -0.78 (-2.35 to 0.79); 0.342 | -0.92 (-2.41 to 0.57); 0.239 |
| ≥25.0 kg/m² | -1.11 (-2.64 to 0.43); 0.172 | -0.95 (-2.68 to 0.78); 0.297 |
| *P* for interaction | 0.111 | 0.158 |

Values are β coefficients with 95% confidence intervals and represent the estimated effect of exercise intervention relative to non-exercise intervention on anxiety and depression scores; negative β values indicate lower symptom scores in the exercise group. Model 1 was unadjusted. Model 2 was fully adjusted for age, sex, smoking exposure (pack-years), COPD duration, COPD severity (FEV1% predicted), hypertension, diabetes mellitus, coronary heart disease, coexisting asthma, and current key COPD medications; baseline HAMA and baseline HAMD were additionally adjusted for the corresponding analyses. BMI categories were defined according to the Asia-Pacific criteria (<23.0, 23.0–24.9, and ≥25.0 kg/m²). *P* for interaction corresponds to the exercise × BMI-category interaction term. HAMA, Hamilton Anxiety Rating Scale; HAMD, Hamilton Depression Rating Scale; CI, confidence interval.

**Supplementary Table 13. Effect of exercise on pulmonary function indices overall and by BMI category**

| **Outcome / Stratum** | **Model 1, β (95% CI); P value** | **Model 2, β (95% CI); P value** |
| --- | --- | --- |
| **MMEF25–75, L/s** |  |  |
| Overall | 0.11 (-0.09 to 0.31); 0.273 | 0.09 (-0.10 to 0.29); 0.357 |
| <23.0 kg/m² | 0.17 (-0.16 to 0.50); 0.320 | 0.15 (-0.20 to 0.51); 0.406 |
| 23.0–24.9 kg/m² | -0.30 (-0.72 to 0.11); 0.169 | -0.34 (-0.74 to 0.06); 0.114 |
| ≥25.0 kg/m² | 0.31 (0.03 to 0.58); 0.033 | 0.34 (0.06 to 0.62); 0.021 |
| *P* for interaction | 0.161 | 0.091 |
| **FVC, L** |  |  |
| Overall | 0.29 (0.01 to 0.57); 0.049 | 0.29 (0.00 to 0.58); 0.052 |
| <23.0 kg/m² | 0.44 (-0.14 to 1.01); 0.150 | 0.47 (-0.18 to 1.12); 0.168 |
| 23.0–24.9 kg/m² | -0.24 (-0.82 to 0.35); 0.435 | -0.29 (-0.88 to 0.29); 0.340 |
| ≥25.0 kg/m² | 0.48 (0.10 to 0.86); 0.016 | 0.59 (0.20 to 0.99); 0.005 |
| *P* for interaction | 0.271 | 0.083 |
| **FEV1, L** |  |  |
| Overall | 0.27 (0.05 to 0.49); 0.017 | 0.24 (0.03 to 0.45); 0.031 |
| <23.0 kg/m² | 0.29 (-0.18 to 0.76); 0.241 | 0.28 (-0.19 to 0.74); 0.264 |
| 23.0–24.9 kg/m² | -0.12 (-0.53 to 0.30); 0.580 | -0.17 (-0.57 to 0.22); 0.394 |
| ≥25.0 kg/m² | 0.47 (0.17 to 0.76); 0.003 | 0.46 (0.16 to 0.77); 0.004 |
| *P* for interaction | 0.186 | 0.166 |
| **FEV1/FVC, percentage points** |  |  |
| Overall | 3.96 (-0.04 to 7.88); 0.051 | 2.27 (-1.23 to 5.76); 0.207 |
| <23.0 kg/m² | 2.05 (-6.01 to 10.11); 0.623 | 0.73 (-5.99 to 7.46); 0.833 |
| 23.0–24.9 kg/m² | 0.13 (-6.63 to 6.90); 0.970 | -0.89 (-7.09 to 5.30); 0.781 |
| ≥25.0 kg/m² | 6.97 (1.19 to 12.76); 0.022 | 3.84 (-1.75 to 9.42); 0.185 |
| *P* for interaction | 0.244 | 0.181 |

Values are β coefficients with 95% confidence intervals and represent the estimated effect of exercise intervention relative to non-exercise intervention on pulmonary function indices; positive β values indicate higher pulmonary function values in the exercise group. Model 1 was unadjusted. Model 2 was fully adjusted for age, sex, smoking exposure (pack-years), COPD duration, COPD severity (FEV1% predicted), hypertension, diabetes mellitus, coronary heart disease, coexisting asthma, and current key COPD medications; the corresponding baseline pulmonary function value was additionally adjusted for each outcome. BMI categories were defined according to the Asia-Pacific criteria (<23.0, 23.0–24.9, and ≥25.0 kg/m²). *P* for interaction corresponds to the exercise × BMI-category interaction term. MMEF25–75, maximal mid-expiratory flow; FVC, forced vital capacity; FEV1, forced expiratory volume in 1 second; CI, confidence interval.

**Supplementary Table 14. Effect of exercise on respiratory muscle strength overall and by BMI category**

| **Outcome / Stratum** | **Model 1, β (95% CI), cmH₂O; P value** | **Model 2, β (95% CI), cmH₂O; P value** |
| --- | --- | --- |
| **MIP** |  |  |
| Overall | 9.30 (0.23 to 18.38); 0.047 | 8.79 (-0.33 to 17.91); 0.062 |
| <23.0 kg/m² | 4.52 (-16.94 to 25.97); 0.683 | 8.17 (-14.92 to 31.26); 0.496 |
| 23.0–24.9 kg/m² | 14.98 (2.68 to 27.28); 0.025 | 13.59 (1.51 to 25.68); 0.038 |
| ≥25.0 kg/m² | 8.77 (-4.76 to 22.31); 0.210 | 7.27 (-6.81 to 21.34); 0.317 |
| *P* for interaction | 0.814 | 0.534 |
| **MEP** |  |  |
| Overall | 5.48 (-4.24 to 15.20); 0.272 | 4.84 (-4.91 to 14.58); 0.333 |
| <23.0 kg/m² | -4.05 (-24.18 to 16.09); 0.697 | -3.25 (-25.40 to 18.90); 0.777 |
| 23.0–24.9 kg/m² | 5.16 (-12.22 to 22.54); 0.566 | 3.38 (-14.08 to 20.84); 0.708 |
| ≥25.0 kg/m² | 11.10 (-3.17 to 25.37); 0.135 | 10.59 (-4.03 to 25.21); 0.163 |
| *P* for interaction | 0.124 | 0.047 |

Values are β coefficients with 95% confidence intervals and represent the estimated effect of exercise intervention relative to non-exercise intervention on respiratory muscle strength; positive β values indicate higher values in the exercise group. Model 1 was unadjusted. Model 2 was fully adjusted for age, sex, smoking exposure (pack-years), COPD duration, COPD severity (FEV1% predicted), hypertension, diabetes mellitus, coronary heart disease, coexisting asthma, and current key COPD medications; baseline MIP and baseline MEP were additionally adjusted for the corresponding analyses. BMI categories were defined according to the Asia-Pacific criteria (<23.0, 23.0–24.9, and ≥25.0 kg/m²). *P* for interaction corresponds to the exercise × BMI-category interaction term. MIP, maximal inspiratory pressure; MEP, maximal expiratory pressure; CI, confidence interval.

**Supplementary Table 15. Effect of exercise on rates of mild and moderate AECOPD overall and by BMI category**

| **Outcome / Stratum** | **Model 1, IRR (95% CI); P value** | **Model 2, IRR (95% CI); P value** |
| --- | --- | --- |
| **Mild AECOPD** |  |  |
| Overall | 0.64 (0.49 to 0.85); 0.002 | 0.64 (0.49 to 0.84); 0.002 |
| <23.0 kg/m² | 0.72 (0.51 to 1.01); 0.074 | 0.70 (0.49 to 1.00); 0.069 |
| 23.0–24.9 kg/m² | 0.68 (0.40 to 1.15); 0.167 | 0.66 (0.38 to 1.15); 0.164 |
| ≥25.0 kg/m² | 0.60 (0.38 to 0.93); 0.029 | 0.57 (0.37 to 0.89); 0.018 |
| *P* for interaction | 0.611 | 0.581 |
| **Moderate AECOPD** |  |  |
| Overall | 0.80 (0.65 to 0.99); 0.047 | 0.81 (0.65 to 1.01); 0.066 |
| <23.0 kg/m² | 0.78 (0.51 to 1.20); 0.275 | 0.75 (0.48 to 1.16); 0.209 |
| 23.0–24.9 kg/m² | 0.90 (0.58 to 1.39); 0.635 | 0.91 (0.59 to 1.40); 0.684 |
| ≥25.0 kg/m² | 0.76 (0.57 to 1.03); 0.089 | 0.73 (0.53 to 1.01); 0.067 |
| *P* for interaction | 0.581 | 0.793 |

Values are incidence rate ratios (IRRs) with 95% confidence intervals and represent the estimated effect of exercise intervention relative to non-exercise intervention on rates of AECOPD during the 4-week intervention period. IRR values <1 indicate lower event rates in the exercise group. Model 1 was unadjusted. Model 2 was fully adjusted for prior AECOPD history, age, sex, smoking exposure (pack-years), COPD duration, COPD severity (FEV1% predicted), hypertension, diabetes mellitus, coronary heart disease, coexisting asthma, and current key COPD medications. BMI categories were defined according to the Asia-Pacific criteria (<23.0, 23.0–24.9, and ≥25.0 kg/m²). *P* for interaction corresponds to the exercise × BMI-category interaction term. Mild AECOPD was defined as an exacerbation requiring intensification of short-acting bronchodilator treatment only; moderate AECOPD was defined as an exacerbation requiring oral corticosteroids and/or antibiotics in addition to short-acting bronchodilators. IRR, incidence rate ratio; AECOPD, acute exacerbation of chronic obstructive pulmonary disease; CI, confidence interval.

**Supplementary Table 16. Effect of exercise on inflammatory cytokines and inflammasome-related markers overall and by BMI category**

| **Outcome / Stratum** | **Model 1, β (95% CI); P value** | **Model 2, β (95% CI); P value** |
| --- | --- | --- |
| **IL-18** |  |  |
| Overall | -1.66 (-8.73 to 5.41); 0.647 | -1.64 (-8.82 to 5.55); 0.657 |
| <23.0 kg/m² | 3.02 (-10.65 to 16.69); 0.671 | 1.83 (-12.76 to 16.42); 0.809 |
| 23.0–24.9 kg/m² | -5.14 (-18.25 to 7.96); 0.451 | -5.74 (-18.68 to 7.21); 0.397 |
| ≥25.0 kg/m² | -1.75 (-12.43 to 8.93); 0.750 | -0.62 (-12.02 to 10.79); 0.916 |
| *P* for interaction | 0.696 | 0.745 |
| **IL-1β** |  |  |
| Overall | -0.43 (-1.66 to 0.80); 0.495 | -0.46 (-1.71 to 0.80); 0.478 |
| <23.0 kg/m² | -0.20 (-2.00 to 1.60); 0.829 | 0.02 (-1.61 to 1.66); 0.977 |
| 23.0–24.9 kg/m² | 0.79 (-1.06 to 2.64); 0.411 | 0.77 (-1.18 to 2.72); 0.449 |
| ≥25.0 kg/m² | -1.22 (-3.30 to 0.86); 0.256 | -1.36 (-3.58 to 0.87); 0.240 |
| *P* for interaction | 0.370 | 0.293 |
| **CASP-1** |  |  |
| Overall | 0.03 (-0.08 to 0.13); 0.612 | 0.03 (-0.08 to 0.14); 0.594 |
| <23.0 kg/m² | 0.05 (-0.19 to 0.30); 0.681 | 0.10 (-0.12 to 0.33); 0.387 |
| 23.0–24.9 kg/m² | 0.03 (-0.11 to 0.17); 0.656 | 0.03 (-0.11 to 0.16); 0.717 |
| ≥25.0 kg/m² | 0.01 (-0.16 to 0.18); 0.872 | 0.03 (-0.16 to 0.21); 0.779 |
| *P* for interaction | 0.772 | 0.785 |
| **NLRP3** |  |  |
| Overall | -0.13 (-0.22 to -0.04); 0.007 | -0.14 (-0.23 to -0.04); 0.007 |
| <23.0 kg/m² | 0.01 (-0.13 to 0.16); 0.839 | 0.00 (-0.15 to 0.15); 0.974 |
| 23.0–24.9 kg/m² | -0.13 (-0.29 to 0.04); 0.163 | -0.13 (-0.29 to 0.03); 0.132 |
| ≥25.0 kg/m² | -0.20 (-0.35 to -0.06); 0.010 | -0.23 (-0.38 to -0.08); 0.005 |
| *P* for interaction | 0.172 | 0.163 |
| **ASC** |  |  |
| Overall | -0.04 (-0.14 to 0.07); 0.473 | -0.04 (-0.15 to 0.06); 0.423 |
| <23.0 kg/m² | -0.01 (-0.26 to 0.24); 0.969 | 0.05 (-0.17 to 0.27); 0.650 |
| 23.0–24.9 kg/m² | 0.05 (-0.11 to 0.21); 0.511 | 0.05 (-0.12 to 0.22); 0.560 |
| ≥25.0 kg/m² | -0.11 (-0.26 to 0.04); 0.177 | -0.12 (-0.27 to 0.04); 0.157 |
| *P* for interaction | 0.390 | 0.521 |

Values are β coefficients with 95% confidence intervals and represent the estimated effect of exercise intervention relative to non-exercise intervention on inflammatory cytokines and inflammasome-related markers; negative β values indicate lower biomarker levels in the exercise group. Model 1 was unadjusted. Model 2 was fully adjusted for age, sex, smoking exposure (pack-years), COPD duration, COPD severity (FEV1% predicted), hypertension, diabetes mellitus, coronary heart disease, coexisting asthma, and current key COPD medications; the corresponding baseline biomarker value was additionally adjusted for each outcome. BMI categories were defined according to the Asia-Pacific criteria (<23.0, 23.0–24.9, and ≥25.0 kg/m²). *P* for interaction corresponds to the exercise × BMI-category interaction term. IL-18, interleukin-18; IL-1β, interleukin-1 beta; CASP-1, caspase-1; NLRP3, NOD-like receptor family pyrin domain-containing 3; ASC, apoptosis-associated speck-like protein containing a CARD; CI, confidence interval.

**Supplementary Table 17. Exploratory mediation analysis of the association between exercise and 6MWD through TFdi in the unadjusted model**

| **BMI category** | **Effect component** | **Estimate (95% CI)** | **P value** |
| --- | --- | --- | --- |
| Overall | Total effect, m | 64.76 (25.49 to 106.65) | <0.001 |
|  | Indirect effect via TFdi, m | 19.02 (1.16 to 37.45) | 0.034 |
|  | Direct effect, m | 45.74 (10.70 to 82.51) | 0.004 |
|  | Proportion mediated, % | 29.37 (2.75 to 64.76) | 0.034 |
| <23.0 kg/m² | Total effect, m | 55.93 (20.43 to 95.99) | <0.001 |
|  | Indirect effect via TFdi, m | 6.60 (-10.44 to 27.85) | 0.486 |
|  | Direct effect, m | 49.33 (18.36 to 82.85) | <0.001 |
|  | Proportion mediated, % | 11.80 (-32.29 to 41.12) | 0.486 |
| 23.0–24.9 kg/m² | Total effect, m | 54.69 (17.09 to 90.43) | 0.004 |
|  | Indirect effect via TFdi, m | 6.96 (-9.67 to 26.05) | 0.404 |
|  | Direct effect, m | 47.73 (12.56 to 77.60) | 0.002 |
|  | Proportion mediated, % | 12.73 (-27.13 to 48.56) | 0.404 |
| ≥25.0 kg/m² | Total effect, m | 59.89 (22.74 to 97.40) | <0.001 |
|  | Indirect effect via TFdi, m | 25.64 (6.97 to 49.91) | 0.002 |
|  | Direct effect, m | 34.25 (1.65 to 66.08) | 0.030 |
|  | Proportion mediated, % | 42.81 (16.59 to 92.53) | 0.002 |

The outcome was 6-minute walk distance (6MWD), the exposure was exercise intervention, and the mediator was diaphragmatic thickening fraction (TFdi). Estimates for the total effect, indirect effect, and direct effect are presented in meters, and the proportion mediated is presented as a percentage. Effects were estimated using nonparametric bootstrap resampling (1,000 iterations), and 95% confidence intervals were derived using the percentile method. BMI categories were defined according to the Asia-Pacific criteria (<23.0, 23.0–24.9, and ≥25.0 kg/m²). This table presents the unadjusted model. 6MWD, 6-minute walk distance; TFdi, diaphragmatic thickening fraction; CI, confidence interval.

**Supplementary Table 18. Exploratory mediation analysis of the association between exercise and 6MWD through TFdi in the fully adjusted model**

| **BMI category** | **Effect component** | **Estimate (95% CI)** | **P value** |
| --- | --- | --- | --- |
| Overall | Total effect, m | 56.07 (18.13 to 97.11) | <0.001 |
|  | Indirect effect via TFdi, m | 19.37 (-0.04 to 42.34) | 0.052 |
|  | Direct effect, m | 36.70 (3.96 to 70.38) | 0.024 |
|  | Proportion mediated, % | 34.54 (-0.10 to 81.96) | 0.052 |
| <23.0 kg/m² | Total effect, m | 48.07 (13.08 to 87.46) | <0.001 |
|  | Indirect effect via TFdi, m | 6.90 (-10.06 to 27.90) | 0.420 |
|  | Direct effect, m | 41.17 (10.04 to 74.74) | 0.002 |
|  | Proportion mediated, % | 14.36 (-42.42 to 51.35) | 0.420 |
| 23.0–24.9 kg/m² | Total effect, m | 47.75 (12.27 to 84.99) | 0.014 |
|  | Indirect effect via TFdi, m | 7.26 (-8.70 to 26.22) | 0.352 |
|  | Direct effect, m | 40.49 (5.42 to 70.49) | 0.016 |
|  | Proportion mediated, % | 15.20 (-28.75 to 68.93) | 0.342 |
| ≥25.0 kg/m² | Total effect, m | 49.76 (14.12 to 86.72) | <0.001 |
|  | Indirect effect via TFdi, m | 17.43 (1.44 to 40.50) | 0.022 |
|  | Direct effect, m | 32.33 (0.75 to 63.48) | 0.044 |
|  | Proportion mediated, % | 35.03 (5.31 to 96.38) | 0.022 |

The outcome was 6-minute walk distance (6MWD), the exposure was exercise intervention, and the mediator was diaphragmatic thickening fraction (TFdi). Estimates for the total effect, indirect effect, and direct effect are presented in meters, and the proportion mediated is presented as a percentage. Effects were estimated using nonparametric bootstrap resampling (1,000 iterations), and 95% confidence intervals were derived using the percentile method. BMI categories were defined according to the Asia-Pacific criteria (<23.0, 23.0–24.9, and ≥25.0 kg/m²). The fully adjusted model was adjusted for baseline TFdi, baseline 6MWD, age, sex, smoking exposure (pack-years), COPD duration, COPD severity (FEV1% predicted), hypertension, diabetes mellitus, coronary heart disease, coexisting asthma, and current key COPD medications. 6MWD, 6-minute walk distance; TFdi, diaphragmatic thickening fraction; CI, confidence interval.
